# Insights into Genetic Diversity Within the Auliekol Cattle Breed Using SNP Genotyping

**DOI:** 10.3390/ani16152346

**Published:** 2026-08-01

**Authors:** Kairat Dossybayev, Talgat Karymsakov, Kanagat Yergali, Altynay Kozhakhmet, Daryn Bekman, Pernebek Sailaubek, Aidar Tapelov, Elena Ciani, Aibyn Torekhanov, Bakytzhan Bekmanov

**Affiliations:** 1 Laboratory of Molecular Genetic Examination, LLP «Kazakh Research Institute of Animal Husbandry and Fodder Production», 51 Zhandosov Street, Almaty 050071, Kazakhstan; doskairat1987.11@gmail.com (K.D.); sailaubek_pernebek@mail.ru (P.S.); tapelov.aidar@gmail.com (A.T.); torehanov.aibyn@mail.ru (A.T.); 2 Laboratory of Bioinformatics and Biostatistics, RSE Institute of Genetics and Physiology, Committee of Science of the Ministry of Science and Higher Education of the Republic of Kazakhstan, 93 Al-Farabi Avenue, Almaty 050060, Kazakhstan; altynaitg@gmail.com; 3 Faculty of Biology and Biotechnology, Al-Farabi Kazakh National University, 71 Al-Farabi Avenue, Almaty 050040, Kazakhstan; 4 Laboratory of Molecular Genetics, RSE Institute of Genetics and Physiology, Committee of Science of the Ministry of Science and Higher Education of the Republic of Kazakhstan, 93 Al-Farabi Avenue, Almaty 050060, Kazakhstan; ergaly.qanagat@gmail.com (K.Y.); boitube1098@gmail.com (D.B.); 5 Department of Biosciences, Biotechnologies and Environment, University of Bari, 4 Via E. Orabona, 70125 Bari, Italy; elena.ciani@uniba.it

**Keywords:** Auliekol cattle, genetic diversity, population structure, SNP markers, *BovineSNP50 BeadChip*, gene flow, genomic relationship matrix, ADMIXTURE, PCA

## Abstract

Understanding the genetic diversity and population structure of farm animals is key to developing effective breeding strategies and conserving genetic resources. The aim of this study was to assess the genetic diversity and population structure of the Auliekol cattle breed based on genotyping using the *BovineSNP50 BeadChip*. This study included 1440 animals from eight farms. Genetic diversity indices (observed and expected heterozygosity), the inbreeding coefficient (F_IS_), and allelic richness were calculated, and population structure was analyzed using PCA, ADMIXTURE, fineSTRUCTURE, and genomic relationship matrix (GRM) methods. The results showed comparable levels of observed and expected heterozygosity across populations (*Ho* = 0.3478–0.3537; *He* = 0.3445–0.3492) and negative F_IS_ values ranging from −0.0211 to −0.0025, suggesting no strong population-level signal of inbreeding. Low F_ST_ (0.007–0.028) and GRM values indicate weak genetic differentiation between populations. PCA and fineSTRUCTURE analyses showed no clear clustering, while ADMIXTURE identified three main genetic components shared across all populations. TreeMix analysis further suggested genetic connectivity among farms, with the Moskalyevskoe population occupying a central position in the inferred network of genetic relationships and migration events. In general, the Auliekol breed is characterized by a high level of genetic coherence and preserved genetic diversity, which creates favorable conditions for its further selective improvement and the preservation of genetic resources.

## Simple Summary

The Auliekol breed is a domestic beef cattle breed of Kazakhstan, formed on the basis of inter-breeding and adapted to local conditions. Assessing the genetic diversity and population structure of this breed is essential for further breeding and preservation of genetic resources. In this study, genetic variability and structure of eight Auliekol cattle populations were analyzed using SNP markers (*BovineSNP50 BeadChip*). The results obtained showed a high level of genetic diversity but weak differentiation between populations and the absence of pronounced inbreeding. PCA, ADMIXTURE, and fineSTRUCTURE analyses supported the presence of a common genetic background and substantial genetic similarity among farms. Moreover, the identified genetic exchange between populations indicates the important role of selection practices and exchange of breeding material. The obtained data can be used to develop effective breeding programs and preserve the Auliekol breed.

## 1. Introduction

Kazakhstan currently maintains over twenty cattle breeds with diverse production profiles, with a total population of approximately 8.83 million head [1]. Both dairy and beef cattle sectors are experiencing concurrent development, driven by growing domestic demand and the expansion of export potential. Notably, beef exports reached approximately 22,000 tons in 2024 [2]. Among the breeds of particular relevance to Kazakhstan’s livestock industry is the Auliekol. The Auliekol is a relatively recently established indigenous breed developed in Kazakhstan (Kostanay region, Moskalyevskoe farm) between 1980 and 1992 [3,4]. The breed was developed through systematic crossbreeding of the Kazakh White-Headed, Charolais, and Aberdeen Angus breeds, starting in the 1960s, followed by rigorous long-term selection based on meat productivity and adaptability to local environmental conditions. The development of the Auliekol cattle breed is shown in Figure S1. The breed received official recognition in 1992 and was subsequently included in the State Register of Animal Breeds of the Republic of Kazakhstan.

Therefore, in accordance with modern scientific requirements, it is imperative to enhance breeding programs for the Auliekol breed. Modern breeding programs and effective conservation management strategies require the study of each breed’s genetic diversity and population structure [5,6]. By studying the genetic characteristics of local cattle breeds, their adaptability, breeding capabilities, and long-term conservation are assessed, which in turn ensures sustainable livestock production. Nowadays, SNP genotyping methods (for example, *Illumina BovineSNP50 BeadChip*) have become useful tools for assessing genome diversity, the level of inbreeding, and genetic relationships between populations for farm animals [6,7,8]. The estimation of allelic richness, private allelic richness, observed and expected heterozygosity, and inbreeding coefficient is often performed using different density SNP data to assess genetic diversity and variability in cattle breeds [9,10,11]. These parameters are commonly used to characterize genetic diversity within and among livestock populations and to support breeding and conservation management decisions. In addition, multidimensional statistical methods such as principal component analysis (PCA), ADMIXTURE, and phylogenetic tree construction are widely used to determine genetic diversity and population structure. These approaches can reveal genetic relationships between populations, their classifications, and the level of genetic mixing as well as contribute to reconstructing historical origins and the evolution of breeds. Furthermore, the genomic relationship matrix method allows us to assess genome similarity between individuals and populations, determine their degree of relatedness, and compare levels of genetic affinity and differentiation. Overall, the analysis of the genetic diversity and population structure of farm animals based on SNP markers provides an important basis for assessing, conserving, and rationalizing their genetic resources in breeding programs.

In recent years, research into genetic features of local cattle breeds of Kazakhstan has been conducted through the widespread use of genomic methods. Thus, in a number of works, the genetic diversity, population structure, and distribution of runs of homozygosity (ROH) in the genome of Kazakh cattle were studied on the basis of SNP markers and data of complete genomic analysis [12,13,14]. In addition, using high-density SNP genotyping and whole-genome sequencing, the population structure, genetic architecture, and selection signatures of some Kazakh cattle breeds were investigated [15,16]. However, the intra-breed genetic structure and the level of genetic diversity of the Auliekol breed in various farms have not yet been sufficiently studied, which emphasizes the scientific and practical importance of a comprehensive genomic analysis of this breed.

The aim of this study is to assess the intra- and inter-population genetic diversity and variability of the Auliekol cattle breed based on genotyping data from 1440 animals obtained using the SNP chip *BovineSNP50 BeadChip*. The results of this study will provide insights into the genetic diversity and population structure of the Auliekol cattle breed and will contribute to the development of scientifically sound strategies for genetic management, breeding, and conservation of this breed.

## 2. Materials and Methods

### 2.1. Experimental Animals

In this study, hair follicle samples were collected from 1440 individuals of Auliekol cattle originating from private farms (Arnash and Bagyt (Karaganda city, Kazakhstan)), (Altyn (Kostanay city, Kazakhstan) and Nurbekov (Almaty city, Kazakhstan)) and limited liability partnerships Timofeevka-Agro, Moskalyevskoe (Kostanay city, Kazakhstan), and UshKarasu (Akmola city, Kazakhstan), as well as from the agricultural enterprise Dievskaya (Kostanay city, Kazakhstan) (Table 1).

**Figure 1:**
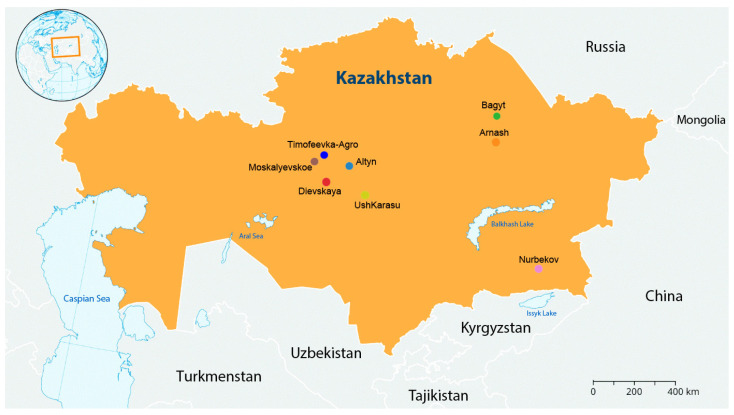
Sampling locations of Auliekol cattle breed.

Figure 1 indicates all sampling locations. Samples were placed in labeled paper envelopes and transported to the laboratory for genomic DNA extraction.

### 2.2. Experimental Methods

#### 2.2.1. Genomic DNA Extraction and Quality Assessment

Collected hair follicle samples were stored in paper envelopes until DNA extraction. Genomic DNA extraction and genotyping on the iScan platform were performed at the Laboratory of Molecular Genetics, Institute of Genetics and Physiology (Almaty, Kazakhstan). DNA was extracted using the DNeasy Blood & Tissue Kit (Qiagen, Hilden, Germany) according to the manufacturer’s protocol. The purity (OD_(260)_/OD_(280)_ ratio) and concentration of the extracted DNA were assessed using a NanoDrop One spectrophotometer (ThermoScientific, Waltham, MA, USA).

#### 2.2.2. SNP Genotyping and Quality Control

SNP genotyping was performed using the Illumina *BovineSNP50 BeadChip* (Illumina, San Diego, CA, USA), yielding 53,218 SNP markers with genomic coordinates mapped to the UMD3.1 bovine genome assembly, as specified in the manufacturer’s manifest file (BovineSNP50_v3_A1.bpm). Quality control (QC) of samples and SNP loci was carried out using the software PLINK (v1.9) [17]. The QC algorithm is presented in Table S1. Only autosomal chromosomes were retained for analysis. SNPs and individuals with a call rate below 95% were excluded. SNPs that significantly deviated from Hardy–Weinberg equilibrium (exact test *p*-value < 1 × 10^−6^) and SNPs with a minor allele frequency (MAF) below 0.01 were also excluded from further analyses. Samples with excessive missing genotypes were additionally excluded using the PLINK --mind parameter. Linkage disequilibrium (LD) pruning was subsequently performed using a sliding window of 50 SNPs, a step size of 5 SNPs, and an r^2^ threshold of 0.2 (--indep-pairwise 50 5 0.2) to obtain a set of largely independent markers for PCA and ADMIXTURE analyses.

#### 2.2.3. Principal Component Analysis

Principal component analysis (PCA) was conducted following QC using 29,308 SNP markers across eight Auliekol cattle populations (*n* = 1438). The analysis was implemented in PLINK (v1.9) [17], and the first two principal components (PC1 and PC2) were used for primary visualization of population structure. Additionally, PC3 was incorporated to generate PC1–PC3 and PC2–PC3 biplots, as well as a three-dimensional PCA plot, which are presented in the Supplementary Materials.

#### 2.2.4. Genetic Diversity Analysis

*Ho* and *He* were calculated for each population based on SNP genotype data. Observed heterozygosity (*Ho*), expected heterozygosity (*He*), and the population inbreeding coefficient (F_IS_) were calculated using Arlequin v3.5 [18]. The F_IS_ coefficient was estimated as the relative difference between expected and observed heterozygosity according to the formula F_IS_ = (*He* − *Ho*)/*He*, where *He* represents the expected heterozygosity under Hardy–Weinberg equilibrium and *Ho* represents the observed heterozygosity [18]. Allelic richness (*Ar*), defined as the number of alleles per locus standardized for sample size, and private allelic richness (*pAr*), representing alleles unique to a given population, were estimated using ADZE [19]. To account for unequal sample sizes among farms, a rarefaction procedure was applied. Rarefaction estimates the expected allelic richness from standardized subsamples of equal size, allowing unbiased comparisons of allelic diversity among populations with different sample sizes.

#### 2.2.5. Population Structure Analysis Using ChromoPainter, fineSTRUCTURE, and ADMIXTURE

Population structure analysis was performed for eight Auliekol cattle populations using ChromoPainter (v0.0.4) and fineSTRUCTURE (v4) [20]. To ensure comparable sample sizes and eliminate the influence of close kinship, genetically related individuals were removed from the original dataset using the --rel-cutoff 0.25 command in PLINK (v1.9) [17]. After filtering, 662 unrelated animals remained from the 1438 original samples. Since this study focused exclusively on the intra-breed structure of the Auliekol breed, a significant reduction in the number of samples after removing related individuals was expected. Thirty-five animals were randomly selected from each of the eight populations (32 samples from the Bagyt farm). The final dataset included 277 samples genotyped for 29,308 SNP markers, with an average genotyping rate of 0.9994. Prior to analysis, SNP genotypes were phased using Beagle (v5.5) [21]. No reference panel was provided, and therefore a Beagle default assumption of 1 cM = 1 Mb was used for recombination rate estimation was used. To estimate the model parameters, 10 iterations of the expectation–maximization (EM) algorithm were performed in ChromoPainter [22]. Sample clustering and population structure estimation were performed in fineSTRUCTURE using a nonparametric Bayesian mixture model implemented based on the Markov chain Monte Carlo (MCMC) algorithm [20,22]. The analysis was performed with default parameters: a total of 200,000 MCMC iterations were run, of which the first 100,000 were discarded as burn-in, with every 1000 iterations saved. In addition, to assess the population structure of the Auliekol cattle breed, an analysis was performed using the ADMIXTURE (v1.3.0) program [23]. The analysis was performed on the same SNP dataset used for the fineSTRUCTURE analysis. ADMIXTURE analysis was performed for K = 2 to 10. For clarity, results for K = 2–5 are presented in the main text, while the complete results for K = 2–10 are provided in the Supplementary Materials (Figure S5). The optimal number of clusters (K) was determined using ADMIXTURE’s built-in 10-fold cross-validation procedure (cv = 10). The K value associated with the lowest CV error was selected as optimal.

#### 2.2.6. Phylogenetic Network Analysis (NeighborNet)

To assess the genetic similarities and differences between the studied animals, a matrix of pairwise genomic distances was constructed using PLINK (v1.9) [17]. The calculation was performed using the --distance square option, which calculates pairwise distances based on the IBS (Identity-By-State) method [24]. Next, the resulting matrix of pairwise genomic distances was used to construct a phylogenetic network using the NeighborNet algorithm [25], implemented in the SplitsTree (v4.11) program [26].

#### 2.2.7. Genomic Relationship Matrix Analysis

To quantify genomic relatedness within and between eight farms representing the Auliekol cattle breed, we used PED/MAP files, where the MAP file contains marker information (chromosome and physical position) and the PED file contains individual genotyping data. The genomic relationship matrix (GRM) was calculated by R version 4.6.0 (R Core Team, Vienna, Austria) using the “VanRaden” [27] method implemented in the “AGHmatrix” package [28], which centers and scales genotypes based on allele frequencies and accounts for missing data. The resulting GRM had dimensions 1438 × 1438, corresponding to the number of animals included in this study. To summarize genomic relatedness at the population level, individual GRM values were aggregated by farm. Mean pairwise genomic relationships were calculated: within each farm (diagonal values) and between farms (off-diagonal values). This produced an 8 × 8 population-level GRM. A heatmap with hierarchical clustering was generated to visualize patterns of genetic similarity and to identify potential clusters of farms sharing common breeding origins or genetic backgrounds.

#### 2.2.8. Analysis of Molecular Variance and Genetic Differentiation

Analysis of molecular variance (AMOVA) as performed on the data described in Section 2.2.3 to VCF format using the --recode vcf option in PLINK (v1.9) [17]. Subsequent analyses were conducted in R version 4.6.0 (R Core Team, Vienna, Austria) using the “vcfR” (v1.16.0), “adegenet” (v2.1.11), and “poppr” (v2.9.8) packages [29,30,31]. AMOVA was performed to estimate the proportion of genetic variance attributable to differences between and within farms [32]. The statistical significance of variance components was assessed using a Monte Carlo permutation test with 10,000 permutations. Pairwise F_ST_ values were calculated using the method of Weir and Cockerham [33] implemented in the “hierfstat” package [34] in R (v4.6.0) and additionally verified using PLINK (v1.9) [17]. 

#### 2.2.9. Inference of Gene Flow Using TreeMix

To estimate gene flow among eight Auliekol cattle populations, data were analyzed using a maximum likelihood graph approach implemented in TreeMix (v1.13) [35]. The analysis was performed on the LD-pruned SNP dataset described in Section 3.1. Allele frequency input data were prepared using the --freq option in PLINK (v1.9) [17]. Migration events ranging from one to five (m = 1–5) were systematically tested. For each value of m, the residual covariance matrix was visually inspected; m = 3 was selected, as it substantially reduced residual magnitude relative to lower m values, while additional migration edges at m = 4–5 did not resolve further notable residuals and lacked clear biological interpretation given the known crossbreeding history of the studied populations. Data visualization was performed in R (v4.6.0) using the built-in plotting functions supplied with the TreeMix (v1.13) [35] software.

## 3. Results

### 3.1. SNP Genotyping and Quality Control of the Auliekol Breed

Genotyping using the BovineSNP50 BeadChip yielded a total of 53,218 SNP markers. Following QC (Section 2.2.2), 1940 SNPs located on sex chromosomes, mitochondrial DNA, or with unknown chromosomal position were excluded, along with 262 SNPs with a call rate below 95%, 3116 SNPs with MAF < 0.01, and 227 SNPs deviating from HWE (*p* < 1 × 10^−6^). The overall genotyping rate across retained SNPs and individuals was 0.998. Two additional samples with excessive missing genotypes were excluded (PLINK --mind). After QC, 47,673 SNPs and 1438 individuals remained for downstream analyses (Table S1). Following LD pruning (r^2^ < 0.2), 29,308 SNPs (genotyping rate 0.999395) remained for PCA and ADMIXTURE analyses.

### 3.2. Genetic Diversity and Phylogeny of Populations

To assess the genetic diversity across populations, the levels of heterozygosity, allelic diversity, and inbreeding coefficients were calculated for the eight populations (Table 2).

The negative or near-zero FIS values indicate a slight excess of observed heterozygosity relative to expected heterozygosity and suggest no strong population-level signal of inbreeding. Observed heterozygosity values ranged from 0.3478 to 0.3537 in the studied Auliekol cattle farms. The results showed that the highest observed heterozygosity value was observed on the Bagyt farm (*Ho* = 0.3537 ± 0.1566), while the lowest value was found on the Moskalyevskoe cattle population (*Ho* = 0.3478 ± 0.1478). The highest expected heterozygosity value was detected on the Altyn population (*He* = 0.3492 ± 0.1430), while the lowest value was observed in the cattle raised on the Nurbekov farm (*He* = 0.3445 ± 0.1453). The inbreeding coefficient (F_IS_) showed negative values across all studied populations, ranging from −0.0211 ± 0.1541 to −0.0025 ± 0.0658. Among all populations, the highest F_IS_ value was observed on the Dievskaya farm. Conversely, the lowest F_IS_ value was recorded on the Nurbekov population, indicating that this group is closest to Hardy–Weinberg equilibrium. The allelic richness (*Ar*) values across the populations ranged from 1.985 to 2.008. The highest allelic richness was recorded on the Timofeevka-Agro population (*Ar =* 2.0079 ± 0.2047), while the lowest value was observed on the Nurbekov farm (*Ar =* 1.9859 ± 0.1840). The remaining populations showed very similar *Ar* values, indicating comparable levels of allelic diversity among them. The calculated private allelic richness (*pAr*) across the populations ranged from 0.0031 to 0.0176. The highest *pAr* value was recorded on the Timofeevka-Agro population (0.0176 ± 0.1114), while the lowest value was observed on the UshKarasu group (0.0031 ± 0.0451). Additionally, the Arnash and Bagyt populations were characterized by a slightly higher number of private alleles compared to the other groups.

### 3.3. Principal Component Analysis (PCA)

**Figure 2:**
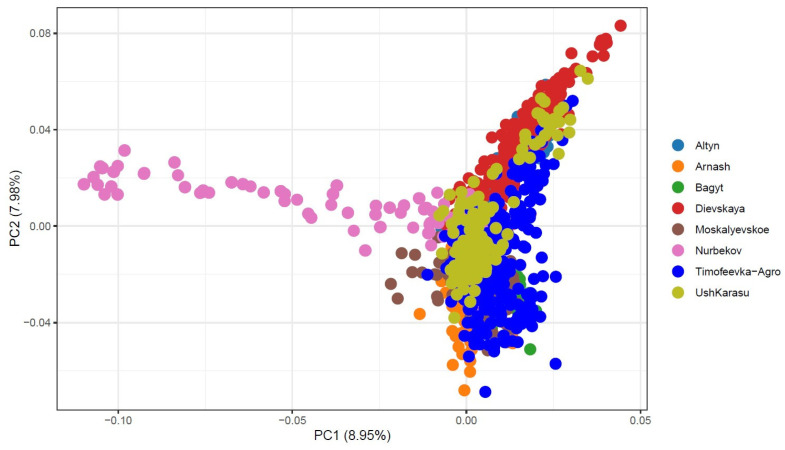
Principal component analysis (PCA) plot based on 1438 Auliekol cattle samples genotyped with the Illumina BovineSNP50 BeadChip.

SNP data obtained from Auliekol cattle were subjected to principal component analysis (PCA) across all studied populations (Figure 2). For the PCA, a total of 29,308 SNPs were evaluated for concordance using PLINK (v1.9) [17] based on the quality control criteria described above. To provide a more detailed view of population differentiation, additional PCA plots combining PCA1–PCA3 and PCA2–PCA3 were constructed, also in 3D figures, as presented in the Supplementary Materials (Figures S2–S4).

The first three components explained a moderate proportion of the total genetic variation (PCA1 8.95%, PCA2 7.98%, PCA3 5.93%), providing a reliable visualization of the spatial relationships among individuals within the breed. In the PCA1–PCA2, animals from most farms clustered tightly together and formed highly overlapping groups. This pattern indicates a high degree of genetic similarity and a shared genetic background. PCA revealed a high degree of overlap among the eight Auliekol cattle populations, indicating limited genetic differentiation. Despite this overall homogeneity, the Nurbekov farm formed a partially separated cluster along PCA1. Additional PCA plots (PCA1–PCA3, PCA2–PCA3, and the 3D plot presented in the Supplementary Materials; Figures S2–S4) further support the observed pattern. In these projections, animals from the Nurbekov farm remain partially separated from the rest of the populations. The broader spread along PCA3 indicates internal genetic variability within this group, potentially reflecting the use of multiple related sire lines or some heterogeneity in the origins of the animals. In contrast, animals from the remaining farms consistently form a single compact cluster with no evidence of pronounced differentiation between geographic regions. The PCA2–PCA3 plot shows only slight differences among several farms (Dievskaya, Moskalyevskoe, Timofeevka-Agro), but these do not form stable or well-defined subgroups and likely represent minor local breeding effects or stochastic genetic drift.

### 3.4. Determination of Population Structure Based on ChromoPainter, fineSTRUCTURE, and ADMIXTURE Analyses

**Figure 3:**
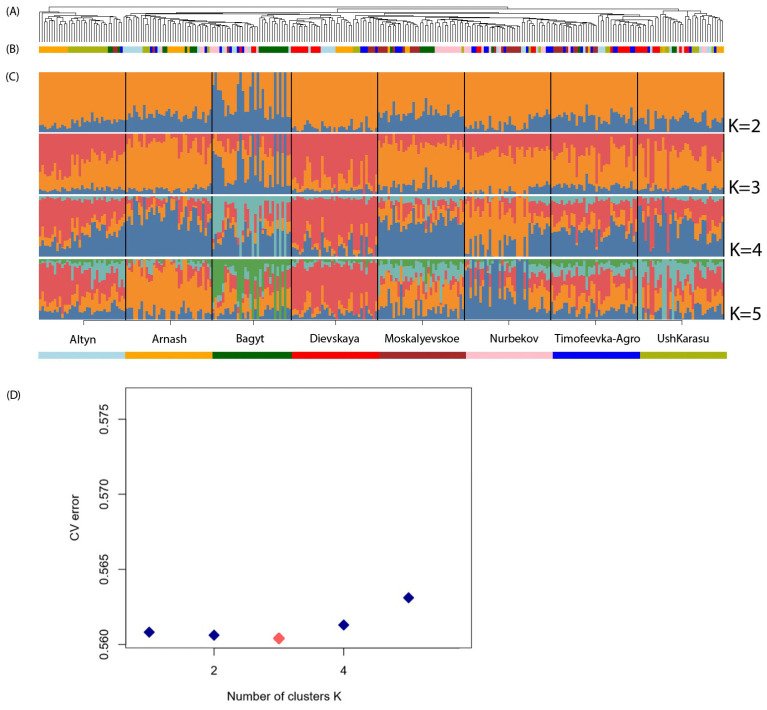
Population structure of eight Auliekol cattle populations inferred from ChromoPainter, fineSTRUCTURE, and ADMIXTURE analyses. (**A**) fineSTRUCTURE dendrogram based on the ChromoPainter coancestry matrix; (**B**) population annotation corresponding to the dendrogram; (**C**) ADMIXTURE clustering results for *K* = 2–5; (**D**) cross-validation (CV) error plot. The optimal K value is highlighted in red.

The haplotype-based analyses performed using ChromoPainter and fineSTRUCTURE revealed weak genetic structuring among the Auliekol cattle populations. The fineSTRUCTURE dendrogram based on the ChromoPainter coancestry matrix demonstrated partial clustering of several individuals; however, clear population-specific separation was not observed, indicating substantial genetic similarity and extensive haplotype sharing among animals from different farms (Figure 3A). Population annotations displayed beneath the dendrogram further confirmed the absence of strict clustering according to population origin (Figure 3B).

Comparable patterns were observed in the ADMIXTURE analysis (Figure 3C). ADMIXTURE was performed for *K* values ranging from 2 to 10, and the optimal number of clusters was determined based on the lowest cross-validation (CV) error (Figure 3D). The minimum CV error was observed at *K* = 3.

At *K* = 2, all populations exhibited highly admixed genetic profiles with no clear differentiation between farms. Although *K* = 3 showed the lowest CV error, the three inferred components were broadly shared across all populations, suggesting a common composite genetic background rather than distinct genetic substructure among contemporary farm populations. It should be noted that, in the absence of reference populations, ADMIXTURE components cannot be directly assigned to specific ancestral sources. With increasing *K* values (*K* = 4–5), no stable or population-specific genetic clusters were observed, further indicating weak genetic differentiation within the breed. At higher *K* values (*K* = 6–10; Supplementary Figure S5), the inferred components become increasingly fragmented, reflecting model overfitting rather than biologically meaningful structure.

### 3.5. NeighborNet Analysis of Studied Populations

**Figure 4:**
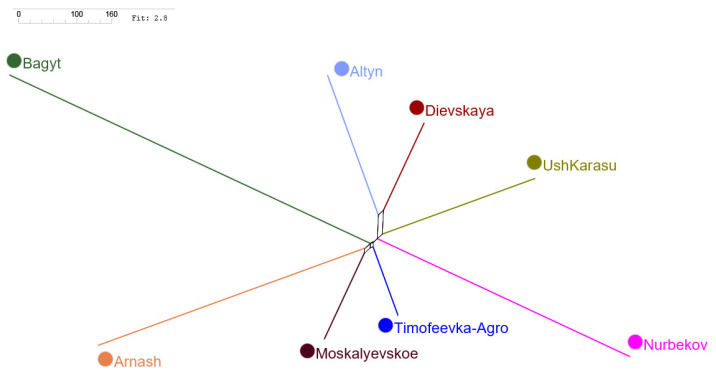
NeighborNet network illustrating genetic relationships among eight Auliekol cattle populations based on genome-wide SNP distances.

The phylogenetic network analysis revealed the genetic relationships among the eight Auliekol cattle populations. All populations were connected through a central node, indicating a common genetic background and a relatively weak population subdivision (Figure 4).

Although the populations were distributed along different branches of the network, the overall structure showed a star-like topology. Among the populations, Bagyt and Arnash were positioned at relatively longer branches from the center, suggesting slightly greater genetic divergence compared to the other populations. In contrast, Altyn, Dievskaya, and Timofeevka-Agro were located closer to the central node, indicating stronger genetic similarity with the overall population structure. Moskalyevskoe and Nurbekov also showed moderate differentiation, whereas UshKarasu occupied an intermediate position between the central cluster and peripheral populations. However, the absence of clearly separated clusters suggests that the populations included in this study do not represent distinct genetic lineages but rather inter-connected components of a single breeding population.

### 3.6. Analysis of the Genomic Relationship Matrix and Genetic Differentiation in the Studied Populations

**Figure 5:**
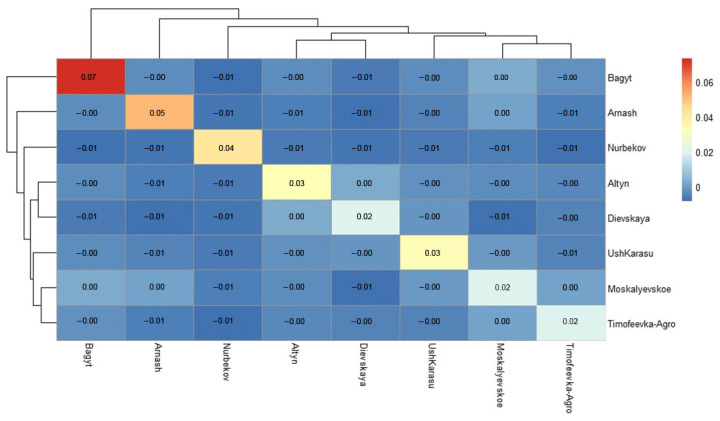
Genomic relationship matrix (GRM) of the Auliekol cattle (eight population).

The genomic relationship matrix (GRM) indicated an overall low level of genomic relatedness between the studied Auliekol cattle populations (Figure 5). Pairwise inter-population GRM values were generally close to zero or slightly negative, suggesting limited excess genomic relatedness between farms. In contrast, higher GRM values were observed within populations (diagonal elements of the matrix), reflecting a greater degree of genomic similarity among individuals from the same farm compared to those from different populations. The inter-population GRM values varied within a narrow range and did not reveal pronounced patterns of relatedness between specific farms. The dendrogram constructed based on the GRM also did not show clearly separated clusters, although some tendency toward grouping of geographically closer populations was observed. It should be noted that GRM values reflect genomic relatedness relative to the allele frequency base used in the analysis and do not directly measure genetic differentiation between populations.

**Figure 6:**
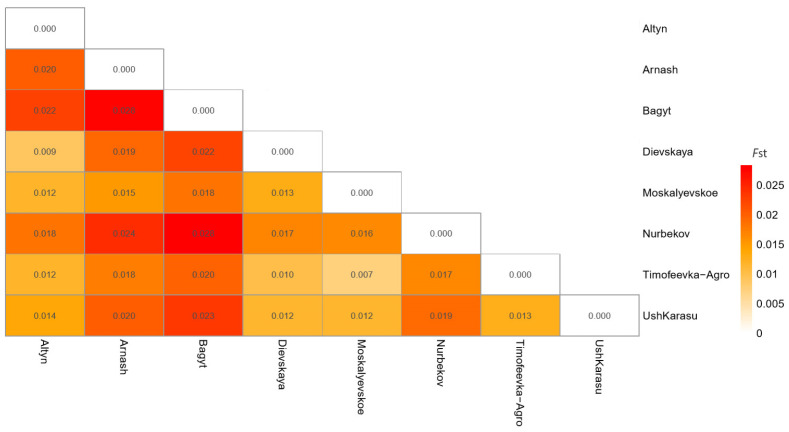
Differentiation of populations based on pairwise F_ST_ for eight populations of Auliekol cattle breed from different geographical locations in Kazakhstan.

Pairwise F_ST_ values among the eight Auliekol cattle farms ranged from 0.007 to 0.028, indicating generally low levels of genetic differentiation between populations (Figure 6). Thus, the farms appear to represent genetically similar populations within a common breeding system.

The lowest pairwise F_ST_ value was observed between Moskalyevskoe and Timofeevka-Agro (F_ST_ ≈ 0.007), indicating very weak genetic differentiation between these farms (Figure 6). Similarly, low values were observed among several other population pairs, particularly among farms located in northern Kazakhstan.

In contrast, the highest F_ST_ values were detected between Bagyt and Arnash, as well as between Bagyt and Nurbekov, reaching approximately 0.028 (Figure 6). Although these values still indicate weak differentiation, they suggest relatively greater genetic divergence compared with other population pairs. This pattern is consistent with the results obtained from the analysis of molecular variance (AMOVA), which showed that 96.922% of the total genetic variation was distributed within populations (intra-population), whereas only 3.078% was attributable to differences among populations (inter-population) (Table 3).

The among-population variance component was statistically significant (*p* < 0.0001), indicating the presence of detectable but limited genetic differentiation between populations.

### 3.7. TreeMix-Based Analysis of Genetic Relationships and Gene Flow

**Figure 7:**
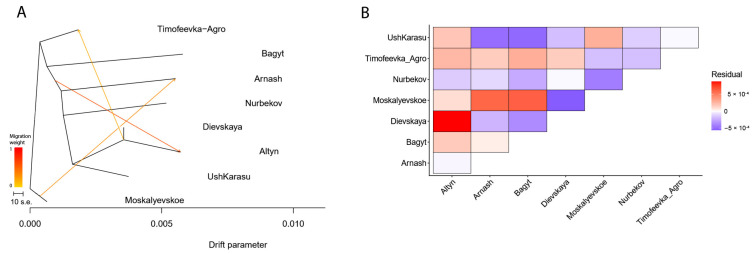
(**A**) TreeMix analysis of eight populations of the Auliekol cattle breed with three migration events (m = 3). (**B**) Residual covariance matrix showing the fit of the TreeMix model to the observed genetic relationships among populations. Color intensity represents the magnitude of residuals, where values close to zero indicate a good fit of the model, and positive and negative residuals indicate minor deviations between observed and expected covariance.

Analysis of the migration plot constructed using TreeMix software, based on the LD-pruned SNP dataset (see Section 3.1), revealed a high degree of genetic proximity among all eight Auliekol cattle populations, consistent with their belonging to a single breed (Figure 7A).

The black branches of the maximum likelihood tree reflect the underlying structure of genetic relatedness among the populations, while the colored arrows indicate putative historical migration events representing gene flow between individual farms. The Moskalyevskoe population is positioned closest to the root of the tree, suggesting relatively lower genetic divergence compared to the remaining populations and allowing it to be considered as the population most closely related to the ancestral genetic core of the breed. Assessment of the goodness of fit of the TreeMix model to the observed data using the residual fit matrix demonstrated that the values of the majority of cells are close to zero, indicating a high degree of correspondence between the constructed model and the empirical data (Figure 7B). Minor positive (red) and negative (blue) deviations observed in individual matrix cells reflect minimal discrepancies between the observed and expected levels of genetic similarity between pairs of populations. Collectively, the results of both analyses demonstrate a high degree of concordance and confirm the common origin of the modern genetic structure of the Auliekol breed. Breed was both by common ancestry. 

## 4. Discussion

This study presents a comprehensive assessment of the genetic structure of the Auliekol cattle breed, sampled from several farms in geographically diverse regions. In this study, the heterozygosity indices in populations of the Auliekol breed of cattle raised on various farms were found to be relatively high. In particular, observed heterozygosity (*Ho*) ranged from 0.3478 to 0.3537, while expected heterozygosity (*He*) ranged from 0.3445 to 0.3492. The results obtained are generally consistent with data from other cattle populations genotyped using the *BovineSNP50 BeadChip*. For example, a genomic study of local cattle populations in Uganda showed that expected heterozygosity values ranged from 0.29 to 0.32 [36]. Similarly, a study conducted on local cattle populations in Mozambique using 50K SNP markers found that *He* values were approximately 0.31 [37]. Similar or slightly lower heterozygosity rates have also been observed in some European breeds belonging to the *Bos taurus* group. For example, in genomic studies of Jersey bulls, the *Ho* and *He* values ranged from 0.33 to 0.34 [38]. Furthermore, in SNP genotyping studies of *Bos taurus* beef breeds such as Aberdeen Angus and Charolais, heterozygosity values ranged from approximately 0.28 to 0.33 [39,40]. Compared with these breeds, the Auliekol population exhibited higher heterozygosity values, suggesting the maintenance of substantial genetic variation within the population. However, such comparisons should be interpreted with caution, as heterozygosity estimates across different studies may be influenced by differences in SNP chip design, ascertainment bias, sample size, filtering criteria, and the genetic background of the populations, potentially affecting the comparability of results. Overall, the relatively high level of heterozygosity may be due to genetic exchange between populations, as well as the long-term effects of both natural and artificial selection. Numerous studies have shown that high levels of genetic diversity in local and adapted cattle populations may be associated with their evolutionary history, adaptation to diverse environmental conditions, and historical gene flow among populations [41,42,43]. In addition, the practice of exchanging breeding animals between farms can also help maintain genetic diversity within populations. At the same time, an alternative explanation for the observed high level of heterozygosity should be considered. In particular, it may be due to the relatively recent origin of the Auliekol breed, which was developed through crossbreeding. It is known that hybridization and crossbreeding of genetically distinct lines can increase the level of heterozygosity by combining different allelic variants within a single population. In this regard, the observed heterozygosity values may reflect not only the preservation of genetic diversity but also the effects of historical crossbreeding that underlie the formation of the breed [44,45,46,47].

In the populations included in this study, allelic richness (*Ar*) ranged from 1.985 to 2.008, while private allelic richness (*pAr*) ranged from 0.0031 to 0.0176. The results indicate a relatively stable level of genetic diversity among the populations. Although private alleles are often considered indicators of population-specific genetic variation and genetic distinctiveness [48], the low and relatively uniform levels of private allelic richness observed in the present study suggest that most genetic variation is shared among farms rather than being restricted to individual populations. This pattern is consistent with the weak population differentiation revealed by F_ST_, AMOVA, PCA, and ADMIXTURE analyses, indicating that the studied populations remain genetically inter-connected and have not accumulated substantial farm-specific genetic variation. The values of the inbreeding coefficient (F_IS_) ranged from −0.0211 to −0.0025 and were negative in all cases. The negative or near-zero F_IS_ values indicate a slight excess of observed heterozygosity relative to expected heterozygosity and suggest the absence of a strong population-level signal of inbreeding [49,50]. This result can probably be partially explained by compliance with the regulatory requirements established by the current rules for the development of livestock breeding in the Republic of Kazakhstan, including regulated ratios between the number of producers and breeding stock, as well as limiting the period of use of breeding bulls (no more than two mating seasons), which helps to reduce the likelihood of inbreeding and prevent the accumulation of inbreeding [51].

Pairwise population differentiation indices (pairwise F_ST_) showed that the level of genetic differentiation among the populations of the Auliekol breed was low (F_ST_ = 0.007–0.028). These values indicate a high degree of genetic similarity among populations and only weak genetic differentiation. According to commonly accepted guidelines in population genetics, F_ST_ values below 0.05 are generally interpreted as evidence of weak population differentiation and limited genetic structure rather than complete genetic homogeneity [52]. The F_ST_ values obtained in the present study are comparable to those reported for other cattle populations. In particular, genome-wide SNP analyses of five indigenous Ethiopian cattle populations (Ambo, Borana, Arsi, Horro, and Danakil) showed an average F_ST_ of approximately 0.01, also indicating a low level of genetic differentiation among populations [53,54]. The low F_ST_ values observed for the Auliekol breed may be attributed to a number of factors. In particular, the exchange of breeding animals between farms and the circulation of breeding stock may have contributed to maintaining the genetic homogeneity of the populations. In addition, the relatively recent formation of the Auliekol breed may also have limited the accumulation of genetic differences between populations. The AMOVA results further support this conclusion, showing that 96.922% of the total genetic variation is maintained within populations, whereas only 3.078% is attributable to differences among populations. Although the among-population component was statistically significant, its magnitude was small, indicating that most genetic diversity is shared across farms rather than partitioned among them. The GRM analysis further indicated limited excess genomic relatedness between farms, with pairwise values generally close to zero. It should be emphasized that GRM reflects genomic relatedness relative to the allele-frequency base used in the analysis and does not directly measure population differentiation [27]. Nevertheless, the low between-population relatedness observed in the present study is consistent with the weak genetic structure revealed by the F_ST_, AMOVA, PCA, and ADMIXTURE analyses.

Additional insights into the relationships among populations were obtained from the TreeMix analysis. The inferred maximum-likelihood tree placed the Moskalyevskoe population closest to the root, suggesting lower genetic divergence relative to the other populations and a closer relationship to the ancestral genetic background of the breed. Furthermore, several inferred migration edges involved Moskalyevskoe, indicating its central position within the network of genetic connections among farms. This observation is consistent with the documented history of the Auliekol breed, which was originally developed at the Moskalyevskoe farm and subsequently expanded to other regions of Kazakhstan. At the same time, TreeMix-inferred migration events should not be interpreted as direct reconstructions of historical animal movements. Rather, they reflect patterns of shared genetic variation that may result from historical and contemporary exchange of breeding material [55]. The residual fit matrix showed that most residual values were close to zero, indicating a good fit of the model to the observed data and supporting the reliability of the inferred relationships among populations. Taken together, the TreeMix results support the view that contemporary Auliekol populations remain genetically inter-connected and share a largely common genetic background while exhibiting only weak population differentiation. Similar patterns have been reported in other cattle populations, where ongoing exchange of breeding animals contributed to reduced genetic differentiation among farms and the maintenance of a relatively homogeneous genomic background [56]. The results of the principal component analysis (PCA) were consistent with previous findings regarding the low level of population differentiation within the Auliekol breed. The results are consistent with data from local cattle populations in Bangladesh, where overlapping clusters in the PCA also indicated weak genetic differentiation between populations [57]. At the same time, the “Nurbekov” population shows some deviation from the main cluster, which may indicate partial genetic differentiation. This result may be attributed to the nature of the breeding program and using similar genetic resources, including semen from centrally distributed artificial insemination sires. In addition, the geographical isolation of this population from other herds likely plays a role, as it may limit the intensity of gene flow and contribute to the development of local genetic characteristics. Similar observations have been reported in a number of studies, in which genetic differences between populations are linked to geographic isolation [8,58,59]. It is emphasized that in livestock breeding, particularly in geographically extensive regions, genetic differentiation among populations is often determined precisely by the spatial distance between them, rather than solely by their breeding history or morphological characteristics. Overall, the fineSTRUCTURE and ADMIXTURE results are consistent with PCA, F_ST_, and GRM data and were consistent with a weak population structure due to common breed origin and subsequent genetic interchange between populations. By contrast, a number of studies have shown that differences in genetic structure among cattle populations can be influenced by different breeding programs. For example, in Holstein cattle, there are differences between populations due to breeding programs in different regions [60]. In this study, the absence of pronounced differentiation between populations of the Auliekol breed may, on the contrary, indicate the use of similar breeding approaches and the exchange of breeding material between farms, which contributes to the preservation of a unified genetic background of the breed. From a breeding and conservation perspective, the observed combination of relatively high genetic diversity, negative F_IS_ values, and weak population differentiation is favorable for the long-term sustainability of the breed. The results suggest that current breeding practices have maintained genetic connectivity among farms while avoiding the development of strong genetic subdivision. Continued genomic monitoring will be important to ensure that genetic diversity is preserved as selection programs progress and breeding objectives evolve.

Several limitations of this study should be acknowledged. The analyses were based on SNP array data, which may be affected by ascertainment bias and do not provide the same resolution as whole-genome sequence data. In addition, more comprehensive assessments of inbreeding and demographic history could be obtained using approaches such as ROH-based inbreeding estimates and effective population size analyses.

## 5. Conclusions

This study provides the first genome-wide assessment of the genetic diversity and population structure of the Auliekol cattle breed across major breeding farms in Kazakhstan. The studied populations showed comparable levels of genetic diversity and only weak genetic differentiation. Most genetic variation was maintained within populations, indicating limited population subdivision among farms. These findings provide a genomic baseline for the management of the Auliekol breed. They support breeding strategies aimed at maintaining genetic variation while limiting population subdivision. The results also provide useful information for future genomic monitoring and conservation of this nationally important cattle breed. This study has several limitations. Pedigree information was not available, and the analyses were based on SNP array data, which may be affected by ascertainment bias. Future studies should integrate pedigree records, whole-genome sequencing, and a broader representation of breeding populations. Such approaches would provide a more comprehensive understanding of the genetic diversity and demographic history of the Auliekol breed while also facilitating the identification of selection signatures and genomic regions associated with economically important traits.

## Figures and Tables

**Table 1:** Description of populations and sample distribution.

Farm Name	Location (Region)	Sample Size (N)	Latitude	Longitude
Altyn	Kostanay region	114	52.22	64.17
Dievskaya	Kostanay region	293	51.98	63.69
Moskalyevskoe	Kostanay region	157	52.20	63.37
Timofeevka-Agro	Kostanay region	308	52.29	63.53
Arnash	Karaganda region	139	49.66	75.81
Bagyt	Karaganda region	74	50.15	76.67
Nurbekov	Almaty region	215	43.54	77.40
UshKarasu	Akmola region	140	51.62	66.48
Total	–	1440	–	–

**Table 2:** Comparative analysis of genetic diversity indicators across Auliekol cattle populations. *Ho*: observed heterozygosity; *He*: expected heterozygosity; *F_IS_*: inbreeding coefficient; *Ar*: allelic richness; and *pAr*: private allelic richness.

Population	n	*Ho*	*He*	F_IS_	*Ar*	*pAr*
Altyn	114	0.3530 ± 0.1487	0.3492 ± 0.1430	−0.0105 ± 0.1104	1.9961 ± 0.1617	0.0034 ± 0.0475
Dievskaya	292	0.3488 ± 0.1450	0.3478 ± 0.1427	−0.0025 ± 0.0658	2.0019 ± 0.1494	0.0034 ± 0.0478
Moskalyevskoe	157	0.3478 ± 0.1478	0.3464 ± 0.1442	−0.0042 ± 0.0872	2.0046 ± 0.1825	0.0090 ± 0.0798
Timofeevka-Agro	308	0.3494 ± 0.1459	0.3473 ± 0.1435	−0.0062 ± 0.0734	2.0079 ± 0.2047	0.0176 ± 0.1114
Arnash	139	0.3513 ± 0.1495	0.3471 ± 0.1441	−0.0117 ± 0.1057	1.9971 ± 0.2091	0.0140 ± 0.0994
Bagyt	74	0.3537 ± 0.1566	0.3459 ± 0.1457	−0.0199 ± 0.1431	2.0023 ± 0.1911	0.0106 ± 0.0863
Nurbekov	214	0.3527 ± 0.1574	0.3445 ± 0.1453	−0.0211 ± 0.1541	1.9859 ± 0.1840	0.0040 ± 0.0521
UshKarasu	140	0.3535 ± 0.1489	0.3477 ± 0.1430	−0.0162 ± 0.1118	1.9903 ± 0.1707	0.0031 ± 0.0451

**Table 3:** Analysis of molecular variance (AMOVA) among eight Auliekol cattle populations based on 47,673 SNP markers.

Source of Variation	Df	Sum of Squares	Variance Component	Percentage of Variation
Among populations	7	373,552.8	258.6438	3.078
Within populations	1430	11,647,317.3	8144.9771	96.922
Total	1437	12,020,870.1	8403.6209	100.000

## Data Availability

The datasets used during the current study are available from the corresponding author on reasonable request.

## Supplementary Materials

The following supporting information can be downloaded at: https://www.mdpi.com/article/10.3390/ani16152346/s1, Supplementary Materials in this article contain the following information: Figure S1. Scheme of the Auliekol cattle breed development through crossbreeding of the Kazakh White-Headed, Aberdeen Angus, and Charolais breeds. The scheme was drawn based on the video “The History of the Creation of the Auliekol Cattle Breed” on the YouTube channel https://www.youtube.com/watch?v=zw0VjMkg0J4 (accessed on 6 June 2026). Table S1. Algorithm for conducting quality control of the dataset for the study of the Auliekol breed. Figure S2. PCA plot based on 1438 Auliekol cattle samples of PC1 vs. PC3; Figure S3. PCA plot based on 1438 Auliekol cattle samples of PC2 vs. PC3; Figure S4. Three-dimensional PCA visualization integrating PC1, PC2, and PC3; Figure S5. Comparison of fineSTRUCTURE and ADMIXTURE analysis results for eight Auliekol cattle populations by K = 2–10. (A) Dendrogram generated from the coancestral matrix; (B) Color annotation of samples according to population assignment; (C) Admixture analysis results (K = 2–10).

## Abbreviations

The following abbreviations are used in this manuscript:

LLP	Limited Liability Partnership
RSE	Republican State Enterprise
SNP	Single Nucleotide Polymorphism
PCA	Principal Component Analysis
GRM	Genomic Relationship Matrix
ROH	Runs Of Homozygosity
QC	Quality Control
HWE	Hardy–Weinberg Equilibrium
MAF	Minor Allele Frequency
LD	Linkage Disequilibrium
IBS	Identity By State

## References

[1] (2025). Bureau of National Statistics of the Agency for Strategic Planning and Reforms of the Republic of Kazakhstan. https://stat.gov.kz/en.

[2] (2024). Bureau of National Statistics of the Agency for Strategic Planning and Reforms of the Republic of Kazakhstan. https://stat.gov.kz/en.

[3] Kineev M., Erdenov B. (2005). Cattle breeds of Kazakhstan.

[4] Danilenko O., Tamarovsky M. (2017). Breeding Auliekolsky cattle in Kazakhstan. Agrar. Sci..

[5] Laikre L., Hoban S., Bruford M., Segelbacher G., Allendorf F., Gajardo G., Rodriguez A., Hedrick P., Heuertz M., Hohenlohe P. (2020). Post-2020 goals overlook genetic diversity. Science.

[6] Liu B., Tao W., Feng D., Wang Y., Heizatuola N., Ahemetbai T., Wu W. (2022). Revealing genetic diversity and population structure of endangered Altay white-headed cattle population using 100K SNP markers. Animals.

[7] Matukumalli L., Lawley C., Schnabel R., Taylor J., Allan M., Heaton M., O’Connell J., Moore S., Smith T., Sonstegard T. (2009). Development and characterization of a high-density SNP genotyping assay for cattle. PLoS ONE.

[8] Vostry L., Vostra-Vydrova H., Moravcikova N., Kasarda R., Cubric-Curik V., Brzakova M., Solkner J., Shihabi M., Moreno J., Spehar M. (2023). Genomic diversity and population structure of the Czech Holstein cattle. Livest. Sci..

[9] Mahar K., Goli R., Chishi K., Ganguly I., Dixit S., Singh S., Choudhary S., Rathi P., Chinnareddyvari C., Diwakar V. (2024). Runs of homozygosity decipher genetic diversity in cattle breed dwelling in the colder regions of the world. Cytogenet. Genome Res..

[10] Wang P., Ou G., Li G., Li H., Zhao T. (2024). Analysis of genetic diversity and structure of endangered Dengchuan cattle population using a single-nucleotide polymorphism chip. Anim. Biotechnol..

[11] Zinovieva N., Dotsev A., Sermyagin A., Deniskova T., Abdelmanova A., Kharzinova V., Sölkner J., Reyer H., Wimmers K., Brem G. (2020). Selection signatures in two oldest Russian native cattle breeds revealed using high-density single nucleotide polymorphism analysis. PLoS ONE.

[12] Beishova I., Dossybayev K., Shamshidin A., Belaya A., Bissembayev A., Khamzin K., Kovalchuk A., Nametov A. (2022). Distribution of homozygosity regions in the genome of Kazakh cattle breeds. Diversity.

[13] Beishova I., Dossybayev K., Shamshidin A., Belaya A., Bissembayev A., Khamzin K., Kovalchuk A., Nametov A. (2022). Population analysis and genetic structure of two Kazakh cattle breeds using 150K SNP. Hayati J. Biosci..

[14] Khamzina A., Yurchenko A., Yudin N., Ibragimov P., Ussenbekov Y., Larkin D. (2024). History, status and genetic characteristics of native cattle breeds from the Republic of Kazakhstan. Vavilovskii Zhurnal Genet. Sel..

[15] Niyazbekova Z., Xu Y., Qiu M., Wang H., Primkul I., Nanaei H., Ussenbekov Y., Kassen K., Liu Y., Gao C.Y. (2025). Whole-genome sequencing reveals genetic architecture and selection signatures of Kazakh cattle. Zool. Res..

[16] Khamzina A., Igoshin A., Muslimova Z., Turgumbekov A., Khussainov D., Yudin N., Ussenbekov Y., Larkin D. (2025). Resequencing composite Kazakh Whiteheaded cattle: Insights into ancestral breed contributions, selection signatures, and candidate genetic variants. Animals.

[17] Purcell S., Neale B., Todd-Brown K., Thomas L., Ferreira M.A., Bender D., Maller J., Sklar P., de Bakker P., Daly M. (2007). PLINK: A tool set for whole-genome association and population-based linkage analyses. Am. J. Hum. Genet..

[18] Excoffier L., Lischer H. (2010). Arlequin suite ver 3.5: A new series of programs to perform population genetics analyses under Linux and Windows. Mol. Ecol. Resour..

[19] Szpiech Z., Jakobsson M., Rosenberg N. (2008). ADZE: A rarefaction approach for counting alleles private to combinations of populations. Bioinformatics.

[20] Lawson D., Hellenthal G., Myers S., Falush D. (2012). Inference of Population Structure using Dense Haplotype Data. PLoS Genet..

[21] Browning B., Tian X., Zhou Y., Browning S. (2021). Fast two-stage phasing of large-scale sequence data. Am. J. Hum. Genet..

[22] Leslie S., Winney B., Hellenthal G., Davison D., Boumertit A., Day T., Hutnik K., Royrvik E., Cunliffe B., Lawson D.J. (2015). The fine-scale genetic structure of the British population. Nature.

[23] Alexander D., Novembre J., Lange K. (2009). Fast model-based estimation of ancestry in unrelated individuals. Genome Res..

[24] Chang C., Chow C., Tellier L., Vattikuti S., Purcell S., Lee J. (2015). Second-generation PLINK: Rising to the challenge of larger and richer datasets. GigaScience.

[25] Bryant D., Huson D.H. (2023). NeighborNet: Improved algorithms and implementation. Front. Bioinform..

[26] Huson D., Bryant D. (2024). The SplitsTree App: Interactive analysis and visualization using phylogenetic trees and networks. Nat. Methods..

[27] VanRaden P. (2008). Efficient methods to compute genomic predictions. J. Dairy. Sci..

[28] Amadeu R., Garcia A., Munoz P., Ferrão L. (2023). AGHmatrix: Genetic relationship matrices in R. Bioinformatics.

[29] Kamvar Z., Tabima J., Grünwald N. (2014). Poppr: An R package for genetic analysis of populations with clonal, partially clonal, and/or sexual reproduction. Peer J..

[30] Knaus B., Grünwald N. (2017). vcfR: A package to manipulate and visualize variant call format data in R. Mol. Ecol. Resour..

[31] Jombart T. (2008). adegenet: An R package for the multivariate analysis of genetic markers. Bioinformatics.

[32] Excoffier L., Smouse P., Quattro J. (1992). Analysis of molecular variance inferred from metric distances among DNA haplotypes: Application to human mitochondrial DNA restriction data. Genetics.

[33] Weir B., Cockerham C. (1984). Estimating F-statistics for the analysis of population structure. Evolution.

[34] Goudet J. (2005). Hierfstat, a package for r to compute and test hierarchical F-statistics. Mol. Ecol. Notes.

[35] Pickrell J., Pritchard J. (2012). Inference of population splits and mixtures from genome-wide allele frequency data. PLoS Genet..

[36] Okwasiimire R., Kugonza D., Ruvinskiy D., Weldenegodguad M., Ghanem N., Makgahlela M., Ginja C., Crooijmans R.P.M.A., Kantanen J., Uimari P. (2025). Genomic insights into the population structure and genetic diversity of Ugandan indigenous cattle. Anim. Genet..

[37] King F., Banga C., Visser C. (2021). Genetic diversity and population structure of three native cattle populations in Mozambique. Trop. Anim. Health Prod..

[38] Srikanth K., Jaafar M.A., Neupane M., Ben Zaabza H., McKay S., Wolfe C., Metzger J., Huson H., Van Tassell C., Blackburn H. (2024). Assessment of genetic diversity, inbreeding, and collection completeness of Jersey bulls in the US National Animal Germplasm Program. J. Dairy. Sci..

[39] McTavish E., Decker J., Schnabel R., Taylor J., Hillis D. (2013). New World cattle show ancestry from multiple independent domestication events. Proc. Natl. Acad. Sci. USA.

[40] Porto-Neto L., Kijas J., Reverter A. (2014). The extent of linkage disequilibrium in beef cattle breeds using high-density SNP genotypes. Genet. Sel. Evol..

[41] Mwai O., Hanotte O., Kwon Y., Cho S. (2015). African indigenous cattle: Unique genetic resources in a rapidly changing world. Asian-Australas. J. Anim. Sci..

[42] Mapiye C., Chikwanha O., Chimonyo M., Dzama K. (2019). Strategies for sustainable use of indigenous cattle genetic resources in Southern Africa. Diversity.

[43] Nyamushamba G., Mapiye C., Tada O., Halimani T., Muchenje V. (2017). Conservation of indigenous cattle genetic resources in Southern Africa’s smallholder areas. Asian-Australas. J. Anim. Sci..

[44] Decker J., McKay S., Rolf M., Kim J., Molina Alcalá A., Sonstegard T., Hanotte O., Götherström A., Seabury C.M., Praharani L. (2014). Worldwide patterns of ancestry, divergence, and admixture in domesticated cattle. PLoS Genet..

[45] Gebrehiwot N., Aliloo H., Strucken E., Marshall K., Al Kalaldeh M., Missohou A., Gibson J. (2021). Inference of Ancestries and Heterozygosity Proportion and Genotype Imputation in West African Cattle Populations. Front. Genet..

[46] Kenny D., Carthy T., Murphy C., Sleator R., Evans R., Berry D. (2022). The association between genomic heterozygosity and carcass merit in cattle. Front. Genet..

[47] Mulim H., Brito L., Pinto L., Ferraz J., Grigoletto L., Silva M., Pedrosa V. (2022). Characterization of runs of homozygosity, heterozygosity-enriched regions, and population structure in cattle populations selected for different breeding goals. BMC Genom..

[48] Allendorf F.W., Luikart G., Aitken S.N. (2012). Conservation and the Genetics of Populations.

[49] Wright S. (1965). The interpretation of population structure by F-statistics. Evolution.

[50] Hartl D., Clark A. (2007). Principles of Population Genetics.

[51] https://adilet.zan.kz/rus/docs/V1900018404.

[52] Wright S. (1978). Evolution and the Genetics of Populations.

[53] Edea Z., Dadi H., Kim S.-W., Dessie T., Lee T., Kim H., Kim J.-J., Kim K.-S. (2013). Genetic diversity, population structure and relationships in indigenous cattle populations. Front. Genet..

[54] Zerabruk M., Li M.-H., Kantanen J., Olsaker I., Ibeagha-Awemu E.M., Erhardt G., Vangen O. (2012). Genetic diversity and admixture of indigenous cattle from North Ethiopia: Implications of historical introgressions in the gateway region to Africa. Anim. Genet..

[55] Slatkin M. (1987). Gene flow and the geographic structure of natural populations. Science.

[56] Upadhyay M., Chen W., Lenstra J., Goderie C., MacHugh D., Park S., Magee D., Matassino D., Ciani F., Megens H. (2017). Genetic origin, admixture and population history of aurochs (*Bos primigenius*) and primitive European cattle. Heredity.

[57] Bhuiyan M., Lee S.H., Hossain S., Deb G.K., Afroz M.F., Lee S.H., Bhuiyan A. (2021). Unraveling the Genetic Diversity and Population Structure of Bangladeshi Indigenous Cattle Populations Using 50K SNP Markers. Animals.

[58] Berihulay H., Li Y., Liu X., Gebreselassie G., Islam R., Liu W., Jiang L., Ma Y. (2019). Genetic diversity and population structure in multiple Chinese goat populations using a SNP panel. Anim. Genet..

[59] Alvarez I., Fernandez I., Traore A., Menendez-Arias N.A., Goyache F. (2021). Population Structure Assessed Using Microsatellite and SNP Data: An Empirical Comparison in West African Cattle. Animals.

[60] Salehi R., Javanmard A., Mokhber M., Alijani S. (2025). Genomic diversity and population structure analysis of four geographically distinct Holstein cattle populations using genome-wide DNA profiling. Arch. Anim. Breed..

