# Supplementary material for: Insights into Genetic Diversity Within the Auliekol Cattle Breed Using SNP Genotyping
Source: Animals (Basel). 2026 Aug 1;16(15):2346. doi: 10.3390/ani16152346 (PMC13463569; doi:10.3390/ani16152346)
Supplement: Supplementary file 1 [file animals-16-02346-s001.zip › animals-4410558-supplementary.pdf]

Insights into Genetic Diversity Within the Auliekol Cattle Breed Using SNP Genotyping

(Supplementary materials)

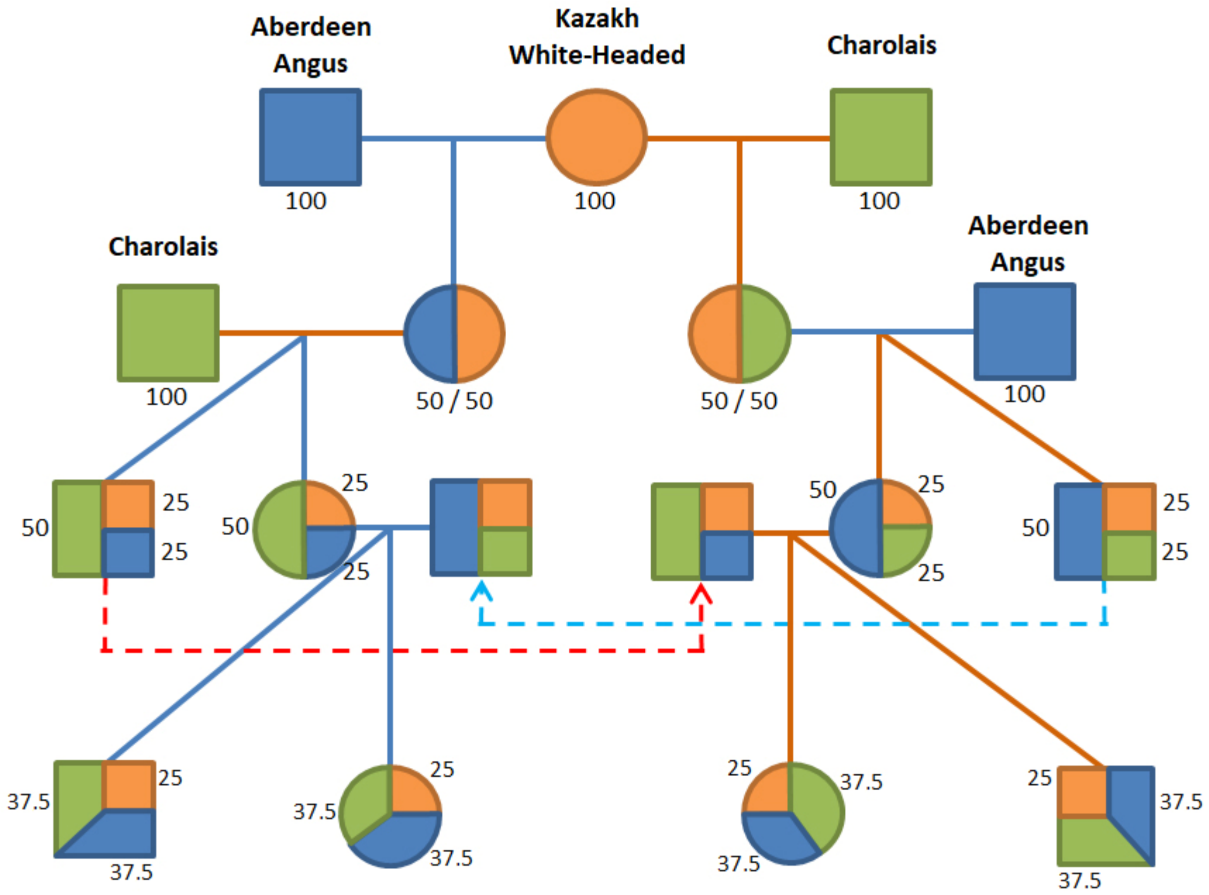

Figure S1. Scheme of the Auliekol cattle breed development through crossbreeding of the Kazakh White-Headed, Aberdeen Angus, and Charolais breeds. The scheme was drawn based on the video "The History of the Creation of the Auliekol Cattle Breed" on the YouTube channel https://www.youtube.com/watch?v=zw0VjMkg0J4 (accessed on 6 June 2026).

Table S1. Algorithm for conducting quality control of the dataset for the study of the Auliekol breed

Dataset	Input SNP	Input sample	Filtration parameters	Output SNP	Output sample
BovineSNP50 BeadChip	53,218	1440	--not-chr X,Y, mtDNA and unknown chromosomal positions	1940	0
BovineSNP50 BeadChip	51,278	1440	--geno 0.05	262	0
BovineSNP50 BeadChip	51,016	1440	--mind 0.05	0	2
BovineSNP50 BeadChip	51,016	1438	--maf 0.01	3116	0
BovineSNP50 BeadChip	47,900	1438	--hwe 1e-6	227	0
BovineSNP50 BeadChip	47,673	1438	--indep-pairwise 50 5 0.2	18,365	0
BovineSNP50 BeadChip	29,308	1438	-	-	-

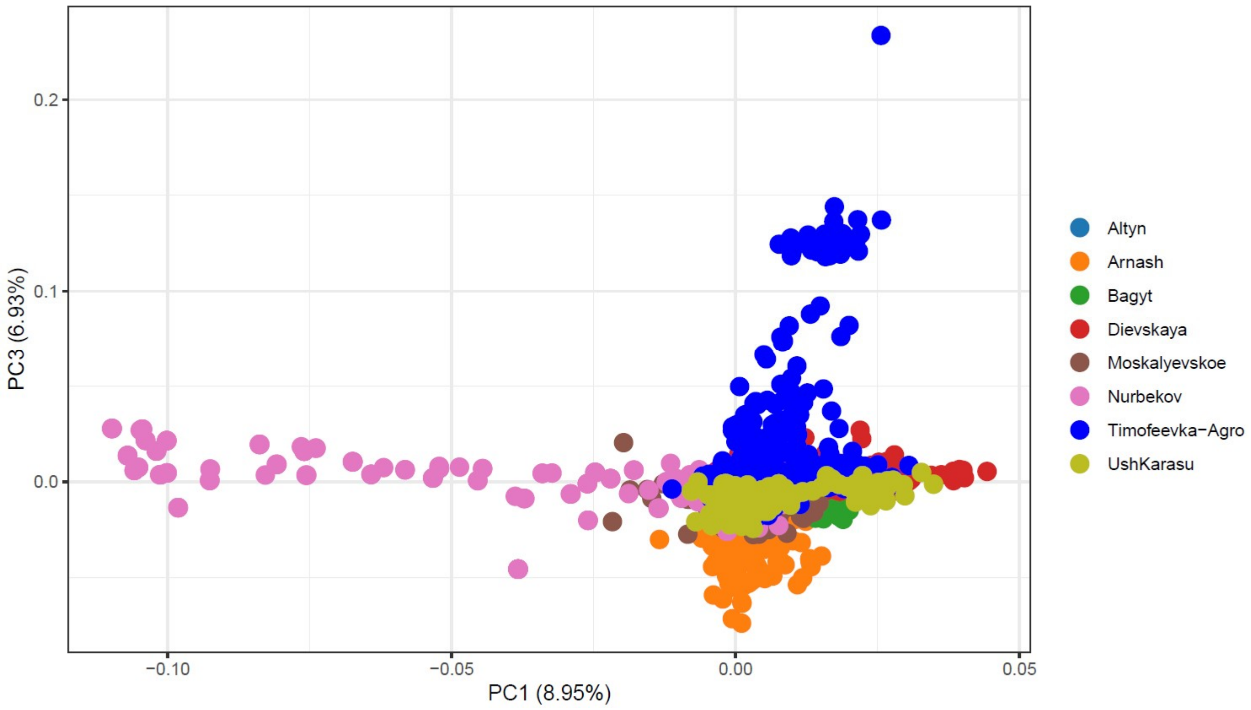

Figure S2. PCA plot based on 1438 Auliekol cattle samples of PC1 vs PC3

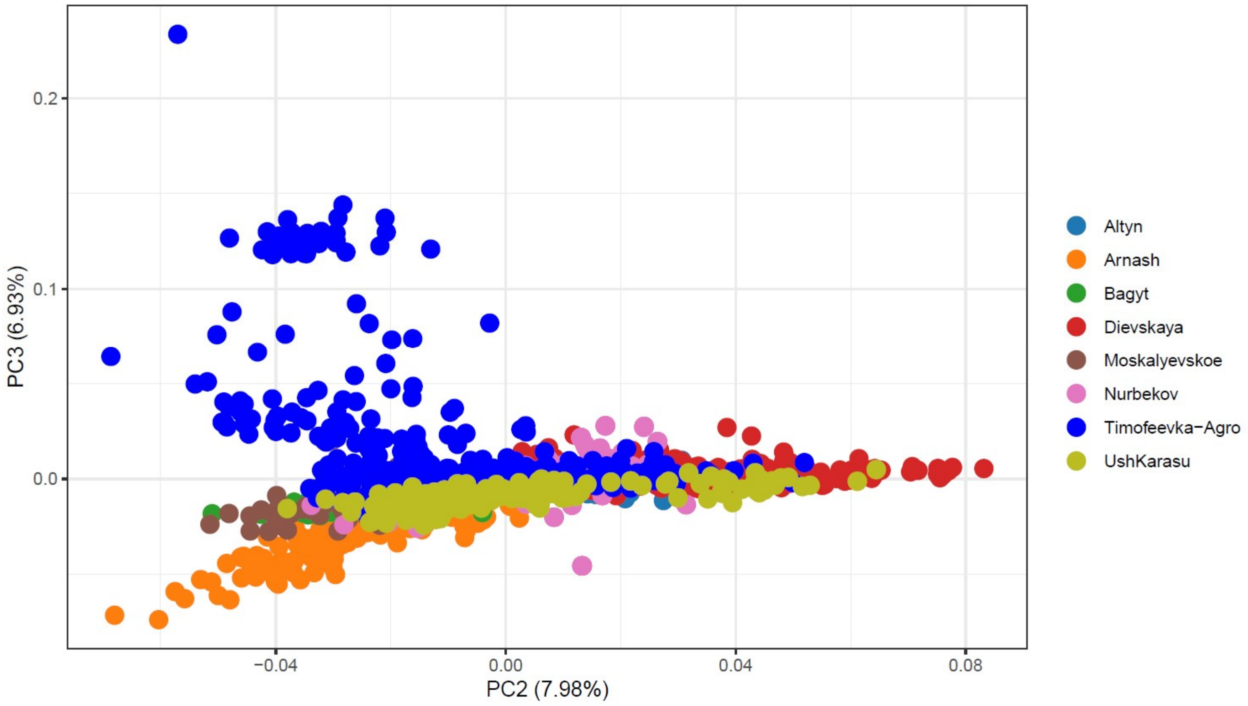

Figure S3. PCA plot based on 1438 Auliekol cattle samples of PC2 vs PC3

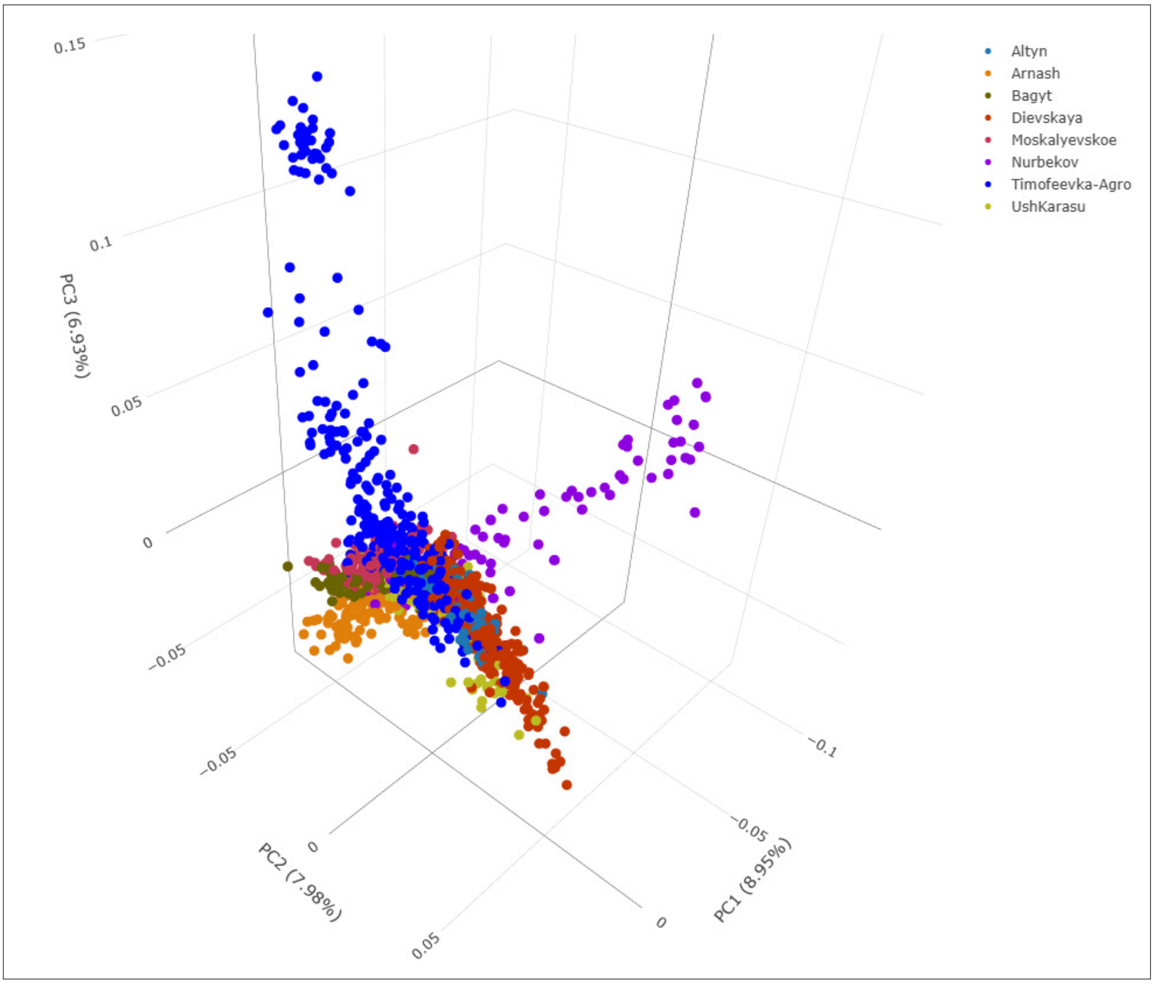

Figure S4. Three-dimensional PCA visualization integrating PC1, PC2, and PC3

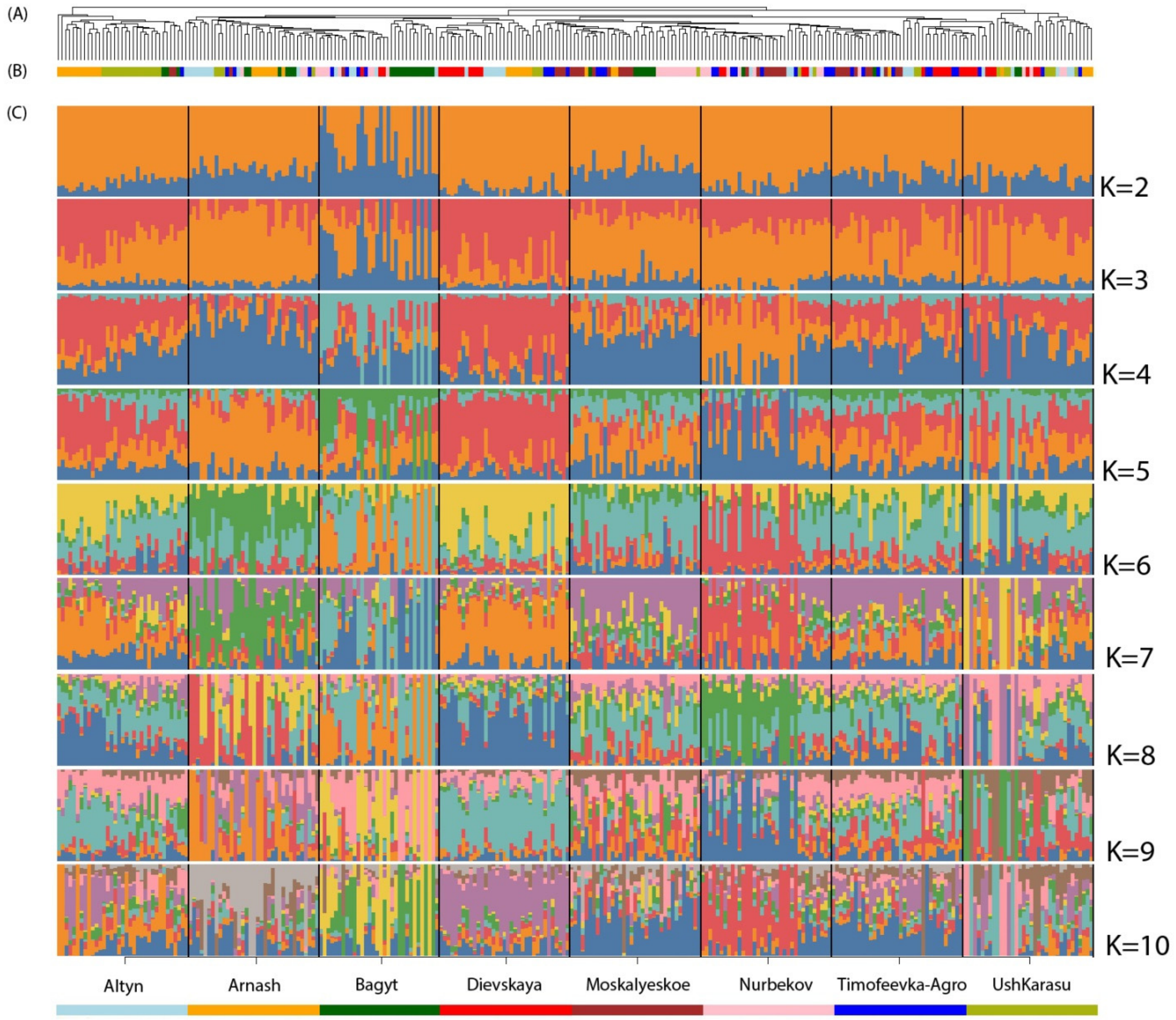

Figure S5. Comparison of fineSTRUCTURE and ADMIXTURE analysis results for eight Auliekol cattle populations by K=2-10. (A) Dendrogram generated from the coancestral matrix; (B) Color annotation of samples according to population assignment; (C) Admixture analysis results (K=2-10)
